# Oral corticosteroid dose changes and impact on peripheral blood eosinophil counts in patients with severe eosinophilic asthma: a post hoc analysis

**DOI:** 10.1186/s12931-019-1056-4

**Published:** 2019-05-03

**Authors:** Charlene M. Prazma, Elisabeth H. Bel, Robert G. Price, Eric S. Bradford, Frank C. Albers, Steven W. Yancey

**Affiliations:** 1 Respiratory Medical Franchise, GSK, Research Triangle Park, 5 Moore Drive, PO Box 13398, Raleigh-Durham, North Carolina 27709 USA; 2 Department of Respiratory Medicine, Academic Medical Center, University of Amsterdam, Amsterdam, Netherlands; 3 Clinical Statistics, GSK, Stevenage, Hertfordshire UK; 4 Respiratory Therapeutic Area, GSK, Research Triangle Park, Raleigh-Durham, NC USA

**Keywords:** Severe eosinophilic asthma, Peripheral blood eosinophil, Oral corticosteroids

## Abstract

**Background:**

An inverse relationship between oral corticosteroid (OCS) dose and peripheral blood eosinophil (PBE) count is widely recognized in patients with severe eosinophilic asthma; however, there are limited data available to quantify this relationship. This post hoc analysis of the SIRIUS study (NCT01691508) examined the impact of weekly incremental OCS dose reductions on PBE counts during the 3–8-week optimization phase of the study.

**Methods:**

SIRIUS was a randomized, double-blind study involving patients with severe asthma (≥12 years old), which included an initial OCS dose optimization phase prior to randomization. Regression analysis assuming a linear relationship between change in OCS dose and change in log (PBE count) during the optimization phase was used to estimate the changes in PBE count following specific decreases in OCS dose.

**Results:**

All 135 patients from the SIRIUS intent-to-treat population were included in this analysis. During the optimization period, 44% (60/135) of patients reduced their OCS dose, with an increase in geometric mean PBE count of 110 cells/μL (200 to 310 cells/μL; geometric mean ratio from beginning to end of the optimization phase: 1.52) recorded in these patients. The model estimated that reduction of daily OCS dose by 5 mg/day led to a 41% increase in PBE count (mean ratio to beginning of optimization phase: 1.41 [95% confidence interval (CI); 1.22, 1.63]).

**Conclusion:**

These data confirmed and quantified the inverse association between OCS dose and PBE count. These insights will help to inform clinicians when tapering OCS doses in patients with severe eosinophilic asthma.

**Electronic supplementary material:**

The online version of this article (10.1186/s12931-019-1056-4) contains supplementary material, which is available to authorized users.

## Background

Eosinophilic airway inflammation, as measured by peripheral blood eosinophil (PBE) count, [1] has been associated with particularly poor symptom control and recurrent exacerbations in a subset of patients with severe asthma. One clinical approach to reduce exacerbations within this patient population is to increase the systemic corticosteroid dose to suppress eosinophilic inflammation. [2] Maintenance oral corticosteroid (OCS) therapy is required in up to 30% of patients with severe asthma; however, physicians are aware of the potential side effects associated with corticosteroid use [3, 4] and, where possible, strive to reduce or remove OCS without compromising symptom control.

While OCS have a broad impact on airway inflammatory cell populations, the efficacy of OCS treatment in patients with severe asthma has been primarily attributed to increased apoptosis of eosinophils. [5] It is widely recognized that an inverse relationship exists between OCS dose and PBE count; however, there are limited data available to physicians that quantify this relationship. The SIRIUS study (NCT01691508) assessed the corticosteroid-sparing effect of mepolizumab in patients with severe eosinophilic asthma. [6] The study included an initial optimization phase, which aimed to define the lowest OCS dose required to maintain asthma control prior to randomization. This post hoc analysis of SIRIUS, examined the impact of incremental OCS dose reductions on PBE counts during the optimization phase.

## Methods

SIRIUS was a 24-week, multicenter, randomized, placebo-controlled, double-blind, parallel-group study in patients ≥12 years of age diagnosed with severe asthma, with a PBE count ≥150 cells/μL at screening or ≥ 300 cells/μL in the prior 12 months. All patients had a documented requirement for treatment with OCS (5–35 mg/day prednisone or equivalent) and high-dose inhaled corticosteroids for ≥6 months prior to study entry, plus additional controller(s) for ≥3 months; there was no exacerbation history requirement. Patients’ asthma control was assessed with the Asthma Control Questionnaire-5 (ACQ-5) at the beginning of the optimization phase (Visit 1), and each subsequent week until randomization. Over the course of the optimization phase (3–8 weeks), patients with controlled or improved asthma status reduced their OCS dose by 5 mg/day each week (if receiving 20–35 mg/day OCS) or 2.5 mg/day each week (if receiving 5–15 mg/day OCS) until they experienced an exacerbation or worsening of asthma control (increase of ≥0.5 points in ACQ-5 score from Visit 1). Following an exacerbation, patients received oral/parenteral corticosteroids at double the current maintenance OCS dose for 3–7 days; thereafter the maintenance OCS dose was increased by one level per the titration schedule. Following worsening of asthma control, patients increased their OCS dose by one level per the titration schedule. All other background medications remained unchanged. In this post hoc analysis we describe the changes over the optimization phase in (1) OCS dose and (2) PBE count in response to OCS dose modification. Additionally, we conducted a regression analysis assuming a linear relationship between change in OCS dose and change in log (PBE count) during the optimization phase. From this model we estimated changes in PBE count following 1 mg/day and 5 mg/day decreases in OCS dose.

## Results

All 135 patients from the SIRIUS intent-to-treat population were included (Additional file 1: Table S1). During the optimization phase, the group median OCS dose decreased from 12.5 mg/day to 10.0 mg/day. In total, 60 (44%) patients decreased their OCS dose over the optimization period, and experienced a corresponding increase in geometric mean PBE count from 200 to 310 cells/μL (increase of 110 cells/μL; geometric mean ratio from beginning to end of the optimization phase: 1.52). Conversely, among the 31 (23%) patients who increased their OCS dose, there was a reduction in geometric mean PBE count from 330 to 200 cells/μL (decrease of 130 cells/μL; geometric mean ratio: 0.58). Forty-four (33%) patients maintained their OCS dose over the optimization phase (reduction in geometric mean PBE count from 240 to 210 cells/μL; decrease of 30 cells/μL; geometric mean ratio: 0.80).

**Fig. 1:**
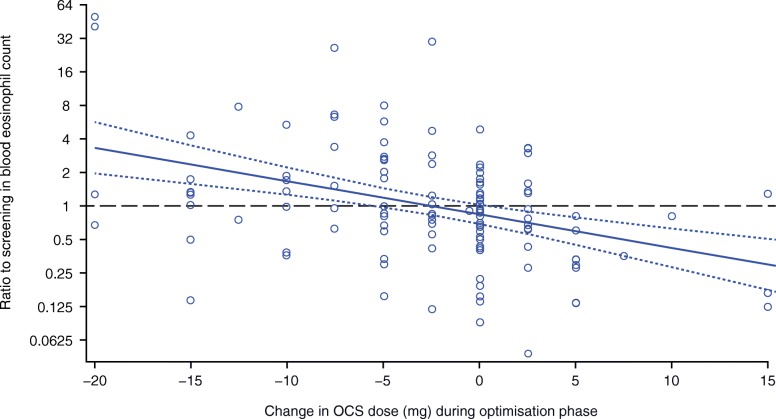
Linear regression of relationship between change in OCS dose and change in blood eosinophil count. Changes in OCS dose and blood eosinophil counts were measured from the beginning to the end of the optimization phase. A ratio greater than 1 reflects an increase, less than 1 reflects a decrease and 1 reflects no change. Solid and dashed blue lines indicate the mean and 95% confidence intervals of this relationship, respectively. The black horizontal line represents no change in blood eosinophil count. Circles represent individual patient values. OCS, oral corticosteroid

Results from the regression analysis confirmed the inverse relationship observed between change in OCS dose and PBE count (Fig. 1). Based on our model, a 1 mg/day reduction in OCS dose resulted in a 7% increase in PBE count during the optimization phase (ratio to beginning of the optimization phase: 1.07 [95% confidence interval: 1.04, 1.10]). Reducing the OCS dose by 5 mg/day resulted in a 41% increase in PBE count during the optimization phase (ratio to beginning of the optimization phase: 1.41 [95% confidence interval: 1.22, 1.63]).

## Discussion

As expected, this analysis demonstrated that a reduction in daily OCS dose led to an increase in PBE count, and an increase in OCS dose results in a decrease in PBE count. Overall, the reduction in group median OCS dose over the optimization phase suggests that some patients may be receiving higher OCS doses than required to control their disease symptoms, while others were on a dose too low to confer control. These data highlight the importance of continually monitoring a patient’s OCS dose to establish, and in some clinical scenarios, reestablish the minimally effective dose for patients receiving chronic OCS therapy and provides an awareness of the correlation between an OCS dose reduction and the impact on PBE. The frequency of real world PBE measurements should be considered on an individual patient basis as various factors may influence PBE such as the initial maintenance OCS dose, the magnitude of change in OCS dose, and the pace of tapering. Additionally, in clinical trials, OCS dose optimization prior to “on-treatment” OCS reduction, may help to mitigate predictable trial effects resulting in large OCS reductions in the placebo arm related to a portion of patients being maintained on a higher OCS dose than that necessary to maintain asthma control.

An increase in PBE count following OCS dose reduction could be indicative of increasing eosinophilic lung inflammation. Prior corticosteroid reduction studies have consistently demonstrated increasing sputum eosinophil counts to be predictive of the development of an exacerbation. [7, 8] Given that sputum eosinophilia (≥2%) has been shown to correlate to blood eosinophil counts ≥150 cells/μL in patients with severe asthma, [9] PBE counts ≥150 cells/μL may also serve as an accessible biomarker for a potential loss of asthma control or future exacerbation.

## Conclusion

Overall, our analysis demonstrated a linear inverse relationship between OCS dose and PBE count, with every 5 mg/day reduction in OCS dose resulting in a 41% increase in PBE count, suggesting that PBE counts are a sensitive marker reflecting changes in OCS dose. These results provide practical guidance to clinicians tapering OCS doses when treating patients with severe eosinophilic asthma.

## Additional file

Additional file 1: **Table S1.** Post hoc analysis of SIRIUS (demographics and clinical characteristics). (DOC 45 kb)

## Abbreviations

ACQ-5: asthma control questionnaire-5
OCS: oral corticosteroid
PBE: peripheral blood eosinophil
