# Supplementary material for: Oral corticosteroid dose changes and impact on peripheral blood eosinophil counts in patients with severe eosinophilic asthma: a post hoc analysis
Source: Respir Res. 2019 May 3;20:83. doi: 10.1186/s12931-019-1056-4 (PMC6499981; doi:10.1186/s12931-019-1056-4)
Supplement: Supplementary file 1 — Table S1. Post hoc analysis of SIRIUS (demographics and clinical characteristics). (DOC 45 kb) [file 12931_2019_1056_MOESM1_ESM.doc]

**Oral corticosteroid dose changes and impact on peripheral blood eosinophil counts in patients with severe eosinophilic asthma: a post hoc analysis (Prazma et al.)**

**Supplementary table. Demographics and clinical characteristics of the SIRIUS ITT population**

|  | **Total population**  **(N=135)** |
| --- | --- |
| Age, years, mean (SD) | 50 (12) |
| Gender, female, n (%) | 74 (55) |
| Body mass index, kg/m^2^, mean (SD) | 28.7 (6.0) |
| Former smoker, n (%) | 53 (39) |
| Duration of asthma, years, mean (SD) | 19 (13) |
| ACQ-5 score at beginning of the optimization phase, mean (SD) | 2.3 (1.14) |
| OCS history in the prior 12 months, n (%) |  |
| Maintenance OCS only | 67 (50) |
| Maintenance OCS plus OCS bursts | 68 (50) |
| OCS dose at beginning of the optimization phase, mg/day, median (range) | 12.5 (5–35) |
| OCS dose ranges at beginning of the optimization phase, n (%) |  |
| 5–10 mg/day | 59 (44) |
| >10–15 mg/day | 26 (19) |
| >15–30 mg/day | 43 (32) |
| >30 mg/day | 7 (5) |
| OCS dose at end of the optimization phase, mg/day, median (range) | 10.0 (5–35) |
| OCS dose ranges at end of the optimization phase, n (%) |  |
| 5–10 mg/day | 69 (51) |
| >10–15 mg/day | 39 (29) |
| >15–30 mg/day | 24 (18) |
| >30 mg/day | 3 (2) |
| Changes in OCS dose from beginning to end of optimization phase, n (%) |  |
| Decreased dose | 60 (44) |
| Maintained (no change in dose) | 44 (33) |
| Increased dose | 31 (23) |
| PBE count at beginning of the optimization phase, cells/µL,  geometric mean (SD Logs) | 230 (1.19) |
| PBE count at end of the optimization phase, cells/µL,  geometric mean (SD Logs) | 270 (1.18) |

ACQ-5, five-item asthma control questionnaire; ITT, intent-to-treat; OCS, oral corticosteroid;
PBE, peripheral blood eosinophil counts; SD, standard deviation
